# User Experiences of and Preferences for Self-Guided Digital Interventions for the Treatment of Mild to Moderate Eating Disorders: Systematic Review and Metasynthesis

**DOI:** 10.2196/57795

**Published:** 2025-01-03

**Authors:** Lauryn Gar-Mun Cheung, Pamela Carien Thomas, Eva Brvar, Sarah Rowe

**Affiliations:** 1 Division of Psychiatry University College London London United Kingdom

**Keywords:** eating disorders, anorexia, bulimia, binge eating, other specified feeding or eating disorder, OSFED, intervention, digital intervention, self-help, systematic review

## Abstract

**Background:**

Digital interventions typically involve using smartphones or PCs to access online or downloadable self-help and may offer a more accessible and convenient option than face-to-face interventions for some people with mild to moderate eating disorders. They have been shown to substantially reduce eating disorder symptoms, but treatment dropout rates are higher than for face-to-face interventions. We need to understand user experiences and preferences for digital interventions to support the design and development of user-centered digital interventions that are engaging and meet users’ needs.

**Objective:**

This study aims to understand user experiences and user preferences for digital interventions that aim to reduce mild to moderate eating disorder symptoms in adults.

**Methods:**

We conducted a metasynthesis of qualitative studies. We searched 6 databases for published and unpublished literature from 2013 to 2024. We searched for studies conducted in naturalistic or outpatient settings, using primarily unguided digital self-help interventions designed to reduce eating disorder symptoms in adults with mild to moderate eating disorders. We conducted a thematic synthesis using line-by-line coding of the results and findings from each study to generate themes.

**Results:**

A total of 8 studies were included after screening 3695 search results. Overall, 7 metathemes were identified. The identified metathemes included the appeal of digital interventions, role of digital interventions in treatment, value of support in treatment, communication at the right level, importance of engagement, shaping knowledge to improve eating disorder behaviors, and design of the digital intervention. Users had positive experiences with digital interventions and perceived them as helpful for self-reflection and mindfulness. Users found digital interventions to be convenient and flexible and that they fit with their lifestyle. Overall, users noticed reduced eating disorder thoughts and behaviors. However, digital interventions were not generally perceived as a sufficient treatment that could replace traditional face-to-face treatment. Users have individual needs, so an ideal intervention would offer personalized content and functions.

**Conclusions:**

Users found digital interventions for eating disorders practical and effective but stressed the need for interventions to address the full range of symptoms, severity, and individual needs. Future digital interventions should be cocreated with users and offer more personalization. Further research is needed to determine the appropriate balance of professional and peer support and whether these interventions should serve as the first step in the stepped care model.

**Trial Registration:**

PROSPERO CRD42023426932; https://www.crd.york.ac.uk/prospero/display_record.php?RecordID=426932

## Introduction

### Background

The lifetime prevalence of eating disorders (EDs) globally is 8.4% for women and 2.2% for men [1]. Health care services in several countries are struggling to meet the demand for ED treatment [2,3], particularly after the impact of the COVID-19 pandemic, which saw an increase in ED symptoms and referrals [4,5]. Health care services are struggling with long waitlists [6], causing specialist ED services to prioritize the treatment of severe cases. This has led to longer durations of untreated EDs [7], which are associated with a worsening of symptoms, such as dangerously low BMI (<13 kg/m^2^) and life-threatening emergencies [2]. This situation leaves individuals with mild to moderate EDs with limited treatment options and at risk of disease progression.

Similarly, people with EDs may have difficulty accessing treatment due to logistical barriers such as lack of transport [8], financial barriers to private psychotherapy [9], and language barriers [8]. For those who struggle to access traditional treatment, digital interventions can offer a more accessible means of managing ED symptoms and distress [10].

Individuals with EDs may also encounter nonstructural barriers to seeking treatment. A substantial issue is the fear of stigma surrounding mental health difficulties and EDs. Individuals with EDs are often believed to be responsible for their mental health difficulty as EDs are commonly perceived to be a “lifestyle choice” as opposed to a mental health issue [11-13]. These attitudes may also be held by health professionals [14], deterring individuals from seeking treatment due to feelings of shame or embarrassment about their ED. This, in turn, contributes to further barriers to receiving professional help [15].

Digital interventions can address structural and logistical barriers to ED treatment by offering greater flexibility and accessibility. They can be accessed via the internet on PCs or mobile devices, often at a low cost, and do not involve lengthy waitlists. Digital interventions can also address nonstructural barriers, such as fear of stigma, by providing a more discrete form of treatment without fear of judgment as opposed to face-to-face approaches.

Digital interventions may be delivered through smartphone apps or websites, and typically adhere to cognitive behavioral models [16]. These models often incorporate psychoeducation, cognitive restructuring, and guided or unguided self-help, and frequently include self-monitoring of symptoms, binges, and compensatory behaviors. Such approaches align with the National Institute for Health and Care Excellence guidelines for ED treatment, which recommends ED-focused cognitive behavioral therapy (CBT) for the treatment of anorexia nervosa, binge ED, and bulimia nervosa [17].

An example of a digital intervention is the Recovery Record mobile phone app [18,19], designed for use either as a self-management tool or tool for clinicians to monitor patient’s thoughts and behaviors between treatment sessions. The app records data on user’s meals, behaviors, feeling, and thoughts and uses gamification of app functions to incentivize logging, rewarding users for every entry. In addition, the app provides meal reminders, positive affirmations, and personalized goals and coping strategies.

An additional example is the web-based cognitive behavioral intervention everyBody Plus [20]. This intervention is intended as a guided self-help intervention for adult women and offers 8 weekly psychoeducation modules covering ED-related topics such as balanced eating, binge eating and purging, improving body image, and dealing with emotions. Similar to the Recovery Record app, everyBody Plus includes functions for logging thoughts and behaviors. However, it specifically focuses on monitoring ED symptoms, such as frequency of binge eating and compensatory behaviors. This intervention represents a more structured approach to digital interventions for EDs, with each session requiring approximately 1 hour to complete and incorporating homework tasks and a group forum.

### Digital Interventions for EDs

Evidence from systematic reviews and meta-analyses support the effectiveness of digital interventions for EDs [21-23]**.** One systematic review and meta-analysis, which included 23 studies, revealed that use of a digital intervention decreased ED behaviors, ED attitudes, and depression after treatment with medium effect sizes that were sustained at follow-up [22]. Another review investigated the efficacy of internet-based programs for EDs and found reductions in shape and weight concern, drive for thinness, and bulimic symptoms with small to medium effect sizes [23].

However, existing research indicates digital interventions for EDs can have high dropout rates. For instance, a study exploring web-based CBT for patients with EDs reported a dropout rate of 37.6% [24]. A systematic review of internet-based treatments for EDs found that dropout rates range from 5.3% to 76.8%, with an average rate of 26.3% [25]. These dropout rates are consistent with digital interventions for other psychological disorders, where dropout rates range from 2 to 83%, with an average of 31% [26].

When comparing dropout rates between digital and face-to-face interventions, digital interventions generally see higher dropout rates [24,27,28]. Specifically, internet-based CBT had a dropout rate of 25% to 30%, whereas face-to-face CBT saw lower dropout rates of 24% to 25% [29]. The higher dropout in digital interventions may be due to engagement challenges [30], while dropout in face-to-face therapy is more likely attributed to therapeutic relationships [31].

Further research is needed to understand why digital interventions are acceptable [32] and effective [22,23] yet have poor treatment adherence, as this limits their potential effectiveness. Examining user experiences and user preferences of digital ED interventions may provide some insights into the factors affecting adherence and engagement.

### Prior Work on User Experience and Preferences

User experience describes the way people use an interactive product [33] and includes the combination of the following components: (1) content, presentation, the esthetic appeal of the product; (2) functionality, the capabilities of the product; (3) interactivity, the way users engage with and navigate through the product; (4) manipulation, the control users have in the product’s features and functions; (5) stimulation, the sensory experience from the product, such as visual and auditory feedback; (6) identification, how users perceive and connect with the product, including their sense of ownership; and (7) perceived user control and evocation, the cognitive responses generated in relation to the product, and their consequences [34, 35]. User experience influences initial commitment to an intervention, therefore has a key role in whether it is adopted [36].

Previous studies suggest users typically find digital ED interventions easy to use and useful [37,38]. Evidence supporting this includes a thematic analysis on 9 participants who used an internet-based CBT intervention, where all participants agreed the intervention was easy to use [38]. However, other users report that certain digital interventions for EDs have poor usability due to counterintuitive interfaces and basic technical malfunctions [39].

Digital interventions are valued for offering confidentiality, privacy, and anonymity, which are substantial factors contributing to their appeal [38]. However, users have raised data privacy concerns and questioned the accuracy and credibility of the information provided [40]. Negative user experiences are often attributed to a lack of personalization and ability to meet individual needs (ie, diagnosis, ED subtype, and stage of treatment) [37,41], and some users have felt overwhelmed by the amount of content and tasks available [37]. While psychoeducational content has been appreciated for maintaining user engagement with the intervention [42], there are concerns that such content may be potentially triggering for some users [37,41]. Many users indicated that their motivation to adhere to the intervention increased when they had an opportunity to speak with a health care professional, as they provided a sense of safety and support [37]. However, a minority of users found the involvement of a professional to be distressing and experienced a sense of surveillance [37,42].

User preferences outline the features and functionalities that individuals seek in interactive products, including aspects such as digital functionality, device types, and content-delivery formats [21]. However, many digital interventions are designed without user input and therefore do not solve problems most relevant to users [39]. By identifying user priorities in advance, interventions can be designed to better meet users’ needs, potentially increasing engagement and commitment to treatment [40].

One study with 722 community-based participants used a web-based survey to investigate preferences for various eHealth functions aimed at treating and preventing ED [40]. The study found that preferences and intentions to use the app were consistent across subgroups. Functions receiving >80% endorsement included clinical support, tailored feedback, strategies to change ED cognitions, screening scales to assess symptoms, ED psychoeducation, and just-in-time intervention prompts. Users showed preference for visual content (such as videos and graphs) over audio and text, and they valued opportunities to customize content delivery (eg, through text, images, videos, or audio recordings). The study found that users preferred fewer motivational pop-ups and reminders, in contrast to other studies, which indicated users valued notifications and reminders because they helped prompt healthier food choices [42] and provided structure for meal timings [19,43]. This suggests users may appreciate notifications that support their recovery efforts but are less receptive to less meaningful notifications simply reminding them to use an app. In addition, food logging emerged as a controversial function as some users desired to continue meal tracking habits as a means of relieving stress and reducing fear of gaining weight, whereas others considered it harmful [43].

While user experiences and user preferences address different aspects of interaction with a product, they are inextricably linked, with each influencing the other. For example, a user’s preference for a food logging function may enhance their experience if they find that it meets their functional needs and provides interactivity. Similarly, a particularly positive user experience with a digital intervention might reveal valued functions and features that users had not previously recognized.

Due to rapid advancements in technology, 32,000 new health apps were introduced from 2017 to 2021 [44,45]. Therefore, an updated systematic review was required to synthesize more recent qualitative research in this area. In addition, prior research on user experience and preferences has been limited by small sample sizes and a lack of sufficient qualitative data for in-depth synthesis and insight [19,37,40-43,46]. By conducting a peer review of existing literature, we aimed to collate and interpret the available data for a broader and more generalizable overview of user experiences and preferences.

### Our Study

This study aimed to investigate user preferences for digital ED interventions and how they are experienced. We systematically reviewed studies using self-help with minimal or no guided support. This review investigated the following questions:

1. What are user experiences of digital interventions for reducing ED symptoms in adults?
2. What are user preferences for digital interventions for reducing ED symptoms in adults?

This review synthesized qualitative studies to allow for an in-depth exploration of attitudes, views, and experiences that are critical to intervention acceptability, outcome, and engagement [47].

### Rationale

A previous systematic review of qualitative research explored users’ experiences of computer-based and book-based guided and unguided self-help for EDs [47]. The review compared the experience of using conventional and computer-based self-help but did not explore user preferences for digital interventions. Researchers searched for studies using either guided or unguided self-help. While 38% (3/8) of the included studies were self-guided interventions, the remaining 50% (4/8) of the studies were fully guided self-help interventions.

## Methods

The methods are reported according to the PRISMA (Preferred Reporting Items for Systematic Reviews and Meta-Analyses) checklist [48] (Multimedia Appendix 1). The protocol was preregistered on PROSPERO on May 19, 2023 (CRD42023426932). Ethical approval was not required for this study.

### Eligibility Criteria

Textbox 1 outlines the specific inclusion and exclusion criteria. We specified a relatively broad criteria, as a preliminary search revealed limited studies on digital interventions for EDs.

**Inclusion and exclusion criteria.**

**Inclusion criteria**

- Study: qualitative studies or mixed methods studies with a qualitative substudy; published or unpublished studies conducted between January 2013 and July 2024, in outpatient and naturalistic settings; any language; any country
- Participants: adults (aged ≥18 y) with eating disorder (ED) symptoms; ED symptoms, either self-reported, subclinical or meeting full diagnosis; use of digital interventions to manage ED symptoms
- Intervention: digitally delivered intervention, delivered via a computer, mobile phone app, smartphone app, or tablet; designed to reduce, manage, or help cope with ED symptoms; primarily a self-help or self-guided intervention; interventions where contact with a clinician, therapist, or support worker was optional may be included; for interventions that include contact with a clinician, therapist, or support worker, they may be included if they adhere to the following: (1) interventions offering contact every session can only be included if 1-way communication is used (eg, weekly personalized feedback with no back and forth conversation) and (2) interventions offering unscheduled 2-way communication (eg, occasional, or drop-in back and forth conversations)

**Exclusion criteria**

- Study: quantitative studies, conference abstracts, prevention studies, and systematic or any other reviews
- Participants: participants below the age of 18 years
- Intervention: interventions targeting a specific subgroup with comorbid physical or mental health disorder (eg, digital interventions for EDs in people with type 2 diabetes); delivered through CD-ROMs and vodcasts; all sessions are fully delivered by a clinician, therapist, or support worker (eg, content from sessions are fully guided by a professional, there is no self-guided activity or learning); scheduled 2-way communication (eg, mandatory weekly back and forth conversation during every session); designed to supplement face-to-face treatment; food or exercise monitoring apps

Although young people may be able to reflect and add valuable insights on digital interventions for EDs, participants below the age of 18 years were excluded due to substantial differences in treatment needs and recommended approaches. For instance, recommended treatment for young people often require family members such as in family-based treatment or anorexia nervosa–focused family therapy for children and young people, while adults are typically treated with individual therapy [17,31]. These differences make it inappropriate to generalize findings between the 2 groups.

Our inclusion criteria did not require participants to have used a digital intervention, only that they were able to provide their perspective on how a digital ED intervention might help them with their ED. Therefore, studies asking participants about the hypothetical use of a digital intervention were included as long as the hypothetical intervention met the inclusion criteria.

While digital interventions improve accessibility by removing waitlists and reducing reliance on trained professionals, guided interventions still depend on funding and professional availability. By excluding guided interventions, we focused on those that fully leverage the key advantage of digital approaches—their ability to address health care barriers independently of professional involvement.

Our scoping search revealed studies before 2013 used outdated technologies (ie, CD-ROMs and vodcasts), so we only included studies from 2013 to 2024 to allow a review of comparable technologies. We excluded prevention studies because we focused on people with existing ED symptoms, rather than the at-risk population.

We focused on mild to moderate EDs, which are likely to exist predominantly within outpatient and naturalistic settings. We avoided inpatient settings that are likely to include more severe and enduring EDs. We did not require participants to have an ED diagnosis or meet a threshold on any ED assessments. This is because people with mild to moderate EDs may choose digital interventions as an early intervention or discrete method of treatment, so may not have been formally assessed for a diagnosis or have subthreshold symptoms.

We did not require a minimum treatment duration (eg, treatment must last at least 4 weeks), nor did we require a minimum number of sessions. These were to ensure that we included users who may have had negative experiences and terminated use prematurely.

We excluded studies targeting specific subgroups with comorbid conditions, as users may have specific needs that would lead to user experiences and preferences better attributed to their comorbid condition rather than their ED.

### Search Strategy

We searched for published and unpublished literature using 6 electronic databases. We used 5 electronic databases that searched for published literature: MEDLINE (OVID), PsycINFO (OVID), CINAHL (EBSCOhost), Embase (OVID), and Web of Science (Core Collection). We used the British Library’s EThOS, a repository for UK PhD theses, to search for unpublished literature.

We built on an existing search strategy [49], with adjustments according to our review questions. Changes included the removal of terms that suggested the digital intervention supplemented face-to-face treatment, outdated technology, and other terms irrelevant to our review questions. A specialist university librarian from University College London was consulted and reviewed the final search strategy.

Search terms captured 3 key concepts, EDs, digital interventions, and user experiences or preferences. Search terms included Medical Subject Headings terms and free text terms (Multimedia Appendix 2 provides the full search strategy).

The initial search using all 6 databases was conducted between April 6 and April 13, 2023. The search was rerun on July 13, 2024, to ensure the inclusion of the most recent research. However, the rerun could not be conducted using EThOS due to its unavailability following a serious ransomware attack.

### Study Selection

We imported search results from each database into EndNote (version 20; Clarivate) [50] for storage and Covidence for screening [51]. Search results were deduplicated in Covidence. Any non-English studies were translated using Google Translate.

The first researcher (LC) initially screened titles and abstracts. An independent second reviewer (PT) screened 100 randomly selected studies at the title and abstract screening stage. A third researcher (EB) randomly selected and screened 50% of all titles and abstracts. Any disagreements or uncertainties between researchers about the inclusion or exclusion of studies were resolved through discussion. If disagreement persisted, a fourth reviewer was involved in the discussion.

Full texts were retrieved and checked each against the eligibility criteria. Reasons for exclusion were noted in Covidence. The primary researcher (LC) completed full-text screening and screening of supplementary materials, followed by the independent reviewer (PT), who also reviewed all studies. A third researcher (EB) randomly selected and screened 50% of all titles and abstracts. Any disagreements or uncertainties were resolved through discussion. If disagreement persisted, a fourth reviewer was involved in the discussion.

The screening process involved repeated comparison of title and abstracts against the inclusion and exclusion criteria (Textbox 1). During the full-text screening, the criteria were repeatedly referred back to after reviewing the introduction, methods, and results sections. Given the variation in digital interventions, ranging from fully unguided to fully guided, researchers LC and PT had frequent meetings to discuss any unforeseen ambiguities. Because of the discussions, the inclusion criteria were refined to address any ambiguities.

### Data Extraction

Data on study characteristics and findings were extracted using a predefined data extraction sheet. Extracted data included study information (authors, publication year, and country), study design, population (sample size, sex, ethnicity, age range, age mean, and SD), ED symptoms, inclusion and exclusion criteria, recruitment and setting, digital intervention (type of technology, contact with clinician, content or aims, and frequency or duration), main findings (user experiences and user preferences), and quality appraisal score.

Data extraction was completed by 2 researchers independently (LC and EB). Any disagreements or uncertainties were resolved through discussion. If disagreement persisted, a third reviewer was involved in the discussion.

### Quality Appraisal

Included studies were critically appraised using the Critical Appraisal Skills Programme (CASP) systematic review checklist [52]. The checklist includes 10 domains: aims, methodology, research design, recruitment strategy, data collection, reflexivity, ethics, analysis, findings, and the value of the research. Individual studies were scored on each domain from 0 to 3. If <25% of the criteria was met, the domain was given a score of 0; if 25% to 49% was met, a score of 1; if 50% to 74% was met, then a score of 2; and if 75% to 100% was met, then a score of 3 was given [53].

Scores from all 10 domains were totaled for each study. The maximum possible overall score was 30. The higher the total score, the higher the quality of the study. A total score of <15 indicated the study was poor quality, 15 to 22.4 indicated moderate quality, and 22.5 to 30 indicated high quality [53].

Quality appraisal was conducted by 2 researchers independently (LC and EB). Any disagreements or uncertainties were resolved through discussion. If disagreement persisted, a third reviewer was involved in the discussion. To be inclusive, studies were not excluded based on quality score or quality category. However, quality category was used to weight findings during data analysis.

### Qualitative Analysis

Included studies were exported into NVivo (version 14; Lumivero) [54] for inductive coding. Any section in the included studies titled “results” or “findings,” including any “results” or “findings” in the abstract or discussion section, acted as our data and were coded. This approach was chosen to prioritize the original authors’ interpretation and synthesis of data, rather than reinterpreting the primary data. By focusing on reported results, we aimed to capture the key themes identified as substantial by the original authors, aligning with the focus of this review to synthesize existing literature rather than reanalyzing primary data. Primary data were not coded because they are rarely made available in electronic databases and accessing them through direct contact with authors could not reliably be guaranteed.

We conducted a thematic synthesis of the data [55] using inductive line-by-line coding, in which each line of data was coded according to its meaning and content [56]. After each line was interpreted, codes were grouped into descriptive themes, meaning concepts that were overlapping or similar from one study to another were combined and reevaluated and similarities and differences in codes were compared across studies [55]. We anticipated the coding stage to involve a comprehensive interpretation of text, which would subsequently inform the development of our own themes in relation to our review questions. We used open coding as opposed to a codebook, no specific themes were expected in advance, rather this approach was to allow the results to emerge from the data itself without preconceptions. Codes and initial descriptive themes were organized using mind maps and tables.

One researcher conducted the initial coding to allow for deep familiarity with data, allowing a better ability to recognize subtle patterns and underlying meanings in the data, and greater consistency in interpretation. Initial descriptive themes were reviewed and discussed with researcher PT, then collaboratively refined with reflexivity in mind until clearly defined themes were apparent. These formed analytical metathemes and subthemes relevant to the review questions and go beyond the content of the data into the interpretation of the key messages. Both researchers developed the final analytical themes.

User experience and user preferences were distinct research questions to ensure a comprehensive exploration of each area. Although these were examined separately, their findings are integrated in the Results section to reflect their substantial overlap and mutual influence.

### Patient and Public Involvement

Two female volunteers with lived experience of ED were recruited to support data analysis. They were recruited using convenience sampling by researchers asking their social circles for volunteers.

A 60-minute online video call was conducted with each individual to discuss descriptive themes and final analytical themes. The video call consisted of a brief explanation on the focus of the review and volunteers’ role and importance of their involvement. The volunteer was presented with a textbox containing the review’s results, the textbox outlined the metathemes and subthemes identified. Each metatheme and subtheme was explained and findings from across the studies were summarized. The researcher then asked questions about the volunteers’ thoughts, whether they resonated with the findings, any disagreements or contrasting opinions they had to the research, and any further thoughts they had that the results did not mention.

Themes were presented to volunteers for feedback, such as the “value of support” theme, which highlighted varied communication functions in digital interventions (eg, direct chat with professionals and group forums). The researcher explained the theme further, for instance, while many users valued these features, some felt pressured or sensed a power imbalance. Volunteers were asked how the themes resonated with their experience of EDs and whether any quotes were misinterpreted. Their lived experiences informed their feedback, helping to improve understanding of the data, reevaluate and reshape themes where needed, and improve the validity of the thematic synthesis. They were compensated with a £20 (US $25) Love2Shop e-gift card for their time.

### Reflexivity

To ensure high-quality qualitative research, consideration of researchers’ views and biases on design and analysis were included [57]. LC is a Chinese British, female postgraduate student, without lived experience of an ED. PT is a White British, female PhD student without lived experience of ED. Both researchers are digital helpline volunteers for an ED charity, so understand the current “treatment gap” in ED service provision in the United Kingdom [3]. LC and PT discussed their respective backgrounds and potential biases that might influence their methodological choices and interpretation of data using the Social GGRRAAACCEEESSS model [58].

During the coding process, reflexivity involved continuous critical reflection on factors that may influence the interpretation of data. Researchers actively considered any of their personal experiences of eating problems, awareness of stereotypes, and the impact of media narratives on EDs. They acknowledged that their understanding of EDs was shaped by Western academic institutions and Western theoretical approaches to mental health and eating behaviors and held this in mind to consider various cultural views on mental health and treatment. Initial codes were discussed among researchers, with open questioning of why certain codes were applied. This reflective dialogue aimed to promote openness and curiosity, allowing for consideration of diverse perspectives and a more balanced, nuanced interpretation of the data.

## Results

### Study Selection

**Figure 1:**
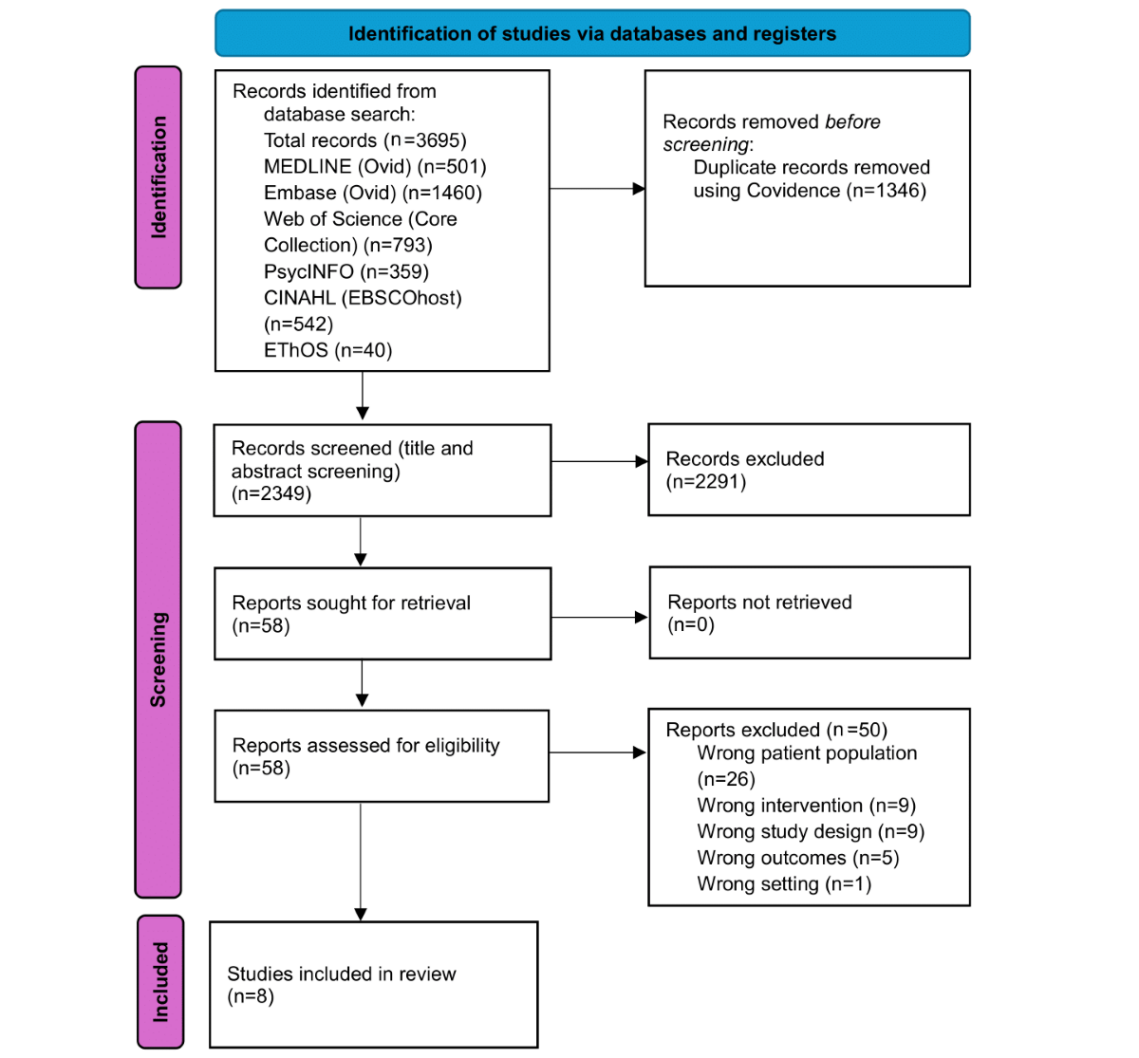
PRISMA (Preferred Reporting Items for Systematic Reviews and Meta-Analyses) flowchart for study selection.

Figure 1 presents a PRISMA flowchart, outlining the screening process that identified the final 8 included studies from 3695 search results. Studies excluded during full-text screening are shown in Multimedia Appendix 2 along with the reasons for exclusion.

Of the 8 included studies, 2 (25%) [59,60] asked participants about their hypothetical preferences for digital interventions rather than preference based on actual use. One study did not require participants to have ED symptoms for inclusion, only self-reported body image issues [60]. However, all participants had a formal ED diagnosis except one. Given body image disturbances are both a risk factor and a symptom of an ED [61], this study was included.

### Study Characteristics

Included studies were published between 2013 and 2022, with sample sizes ranging from 7 to 24 participants and a sample age range of 18 to 67 years. Of the 8 studies, 5 (63%) provided details on the mean sample age, with an average mean age of 35.5 years. Overall, 75% (6/8) of the studies included all-female samples, with only 4 (4%) male participants across all studies. Of the 8 studies, 3 (38%) described the sample’s ethnicities, all of which were predominantly Caucasian or White (n=29, 59%), with remaining ethnicities including Asian (n=6, 12%), African American (n=5, 10%), Pacific Islander (n=3, 6%), Mixed (n=2, 4%), and Eastern European (n=1, 2%).

All included studies were conducted in high-income countries [62-64]: United States (3/8, 37%), United Kingdom (3/8, 37%), and Australia (2/8, 25%). Studies were conducted in community settings (5/8, 63%), or both community and specialist settings (3/8, 37%). Participants were recruited through universities (2/8, 25%), universities and the community (3/8, 37%), or solely through the community (3/8, 37%).

Table 1 outlines study characteristics and intervention details for each study. Digital interventions used include smartphone apps (4/8, 50%), web-based interventions (2/8, 25%), or no specific digital intervention (2/8, 25%). ED symptoms were based on self-report (5/8, 63%), a screening assessment (2/8, 25%), or either self-reported symptoms or assessment (1/8, 13%).

**Table 1:** Table1. Study characteristics and digital intervention details.

Study; country	Study design	Sample details	Digital intervention	Inclusion and exclusion criteria	CASP^a^ quality category^b^
Jafari [65], 2021; United States	Mixed methods study The University of North Carolina at Chapel Hill students recruited via email advertisement Community setting	N=24 (100%) female 18-30 years (mean and SD not specified) White (n=12), African American or Black (n=3), Asian or Asian American (n=4), Hawaiian or Pacific Islander (n=3), mixed race or other (n=2), Hispanic and Latino (n=1)	“Am I Hungry?” Smartphone mobile app No contact with clinician Aimed to help users end restrictive dieting and resolve mindless and emotional eating using self-guided mindfulness exercises Frequency not stated	Inclusion criteria: female, aged 18-30 years, not currently dieting, own and use a smartphone, reported ≥2 binges within the past 28 days, willing to use an app for 4 weeks	High
Yim et al [59], 2020; United Kingdom	Qualitative substudy Sample recruited through 15 NHS^c^ Foundation Trusts, national and regional ED^d^ charities, King’s College London email circulars, social media, and word of mouth Community and specialist outpatient settings	N=12 (100%) female 21-60 years (mean 42.2, SD 13.7) Ethnicity not specified	everyBody Plus Web-based intervention Weekly one-way communication Feedback from a psychologist and access to an online discussion board Aimed to support positive body image, support balanced eating, teach alternative behavioral strategies, and enhance self-confidence Included readings, recording symptoms, binges and compensatory behaviors, behavioral exercises, and a journal 8 weekly sessions	Inclusion criteria: BN^e^, BED^f^, or OSFED^g^ symptoms seeking outpatient treatment Exclusion criteria: BMI <18.5 kg/m^2^, in need for inpatient ED treatment due to severity, psychiatric comorbidity needing treatment in its own right; or on antidepressant medication	High
Juarascio et al [46], 2015; United States	Qualitative study Sample recruited from the Philadelphia community, or referred by clinicians, or a behavioral weight loss program Community and specialist setting	N=11 (91%) female 25-67 years (mean 42.81, SD 13.85) Caucasian (n=7), African American (n=2) unknown (n=2)	Intervention had no name Smartphone mobile app (a prototype) No contact with clinician To reduce binge eating symptoms using food, emotion, and binge logging, automated data entry, CBT^h^-based self-help learning modules, coping strategies, and data visualization Frequency not stated	Inclusion criteria: met criteria for BED, subthreshold BED, or had a history of binge eating	Moderate
McClay et al [66], 2013; United Kingdom	Qualitative substudy Community-based recruitment (advertisements in mental health websites, newsletters, and magazines. Posters in public places and flyers put into university orientation packages) Community setting	N=9 (100%) female 28-50 years (mean 33.9, SD not stated) Ethnicity not specified	Overcoming Bulimia Online Web-based intervention Weekly contact with a support worker (Support was deemed optional as support time varied highly and relied on participant engagement with support) Aimed to change users’ thoughts, feelings, and behavior with regard to food. Modules included psychoeducation, problem-solving, and planning. Each module included homework. Participants recorded mood, binging, and vomiting 8 sessions	Inclusion criteria [67]: aged ≥16 years, resident in the United Kingdom, BMI ≥18.5, met BN or EDNOS^i^ criteria, established using the Eating Disorders Examination [68] Exclusion criteria: currently participating in any other treatment-based ED research, self-reported drug or alcohol addiction, take street drugs daily or weekly, and no regular access to broadband with speakers or headphones	High
Linardon et al [69], 2022; Australia	Mixed methods study Advertisement distributed on the author’s open-access psychoeducational website for EDs [70] Community setting	N=14 (79%) female 19-47 years (mean 32.71, SD 8.26) Caucasian (n=10) Asian (n=2), Eastern European (n=1), Hispanic (n=1)	Break Binge Eating App Smartphone mobile No contact with clinician Included psychoeducation, self-monitoring, learning healthier coping strategies. and body image Frequency not stated	Inclusion criteria: self-reported at ≥1 objective binge eating episode in past 28 days	Moderate
Yim et al [71]**,** 2021; United Kingdom	Qualitative study Recruited sample with posters, clinicians’ referrals, and university circular emails at South London and Maudsley ED Outpatient clinic and King’s College London Community and outpatient setting	N=15 (100%) female Age not specified Ethnicity not specified	No specific digital intervention referred to Not applicable No contact with clinician Participants interviewed on ED needs and barriers and facilitators for online self-help programs 4×30–60-minute sessions	Inclusion criteria [20]: female participants aged ≥18 years. Self-identified as experiencing BN, BED, or a related subthreshold binge and purge ED Exclusion criteria [20]: BMI <18.5 kg/m2, patients in need for inpatient eating disorder treatment due to the severity of the disorder, patients with substantial psychiatric comorbidity needing treatment in its own right, patients on antidepressant medication who have not been on a stable dose for at least 4 weeks, and patients not eligible for treatment coverage by health insurances or NHS due to low symptoms severity	Moderate
Jarman et al [60], 2022; Australia	Qualitative Recruited from a larger project (Web-Based Interventions to Reduce Eating Disorders [WIRED]) via posts on the project’s social media accountsCommunity setting	N=7 (100%) female 21-33 years (mean 25.83, SD=5.34) Ethnicity not specified	No specific digital intervention used Not applicable No contact with clinician Participants interviewed on their needs and preferences for app-based eating disorder interventions Participants discussed user experiences and preferences hypothetically Frequency not stated	Inclusion criteria: self-identified lived experience of ED or substantial body image issues	High
Nitsch et al [72], 2016; United States	Engagement and iterative usability study Web-based and print advertisements in the San Francisco Bay Area and on the campus of a large private university Community setting	N=9 (100%) female 18-25 years(mean and SD not specified) Ethnicity not specified	Student Bodies-Eating Disorders Web-based program and mobile app Unscheduled contact with a personal coach, plus an online, asynchronous guided discussion group [73] Aimed to reduce disordered eating behaviors, improve body image, and support the development of coping skills 40 ×10-minute daily sessions.	Inclusion criteria: a positive response to ≥2 out of 5 on the SCOFF^j^ questionnaire^k^ [74] Exclusion: lack of English language fluency, hearing impairments, and involvement in any depression or anxiety intervention research study	Moderate

^a^CASP: Critical Appraisal Skills Programme.

^b^CASP scores indicate study quality, with a score <15 indicating poor quality, 15 to 22.4 indicating moderate quality, and 22.5 to 30 indicating high quality.

^c^NHS: National Health Service.

^d^ED: eating disorder.

^e^BN: bulimia nervosa.

^f^BED: binge eating disorder.

^g^OSFED: other specified feeding or eating disorder.

^h^CBT: cognitive behavioral therapy.

^i^EDNOS: eating disorder not otherwise specified.

^j^SCOFF: Sick, Control, One Stone, Fat, Food.

^k^The SCOFF questionnaire is a widely used and validated screening tool for eating disorder symptoms.

Digital interventions involved no contact with a clinician or support worker (3/8, 37%) or minimal contact with a clinician (3/8, 37%). Digital interventions with minimal contact with a clinician encapsulate those which fall within the spectrum from guided to unguided, but still meet our inclusion and criteria. The study by Yim et al [59] was included due to the one-way nature of contact, the study by McClay et al [72] was included as the support was deemed optional due to reported high variability in support time due to reliance on the participant engaging with support, and the study by Nitsch et al [72] was included as contact was unscheduled and contact through discussion group was optional (Table 1 provides further details on contact with professionals).

### Quality Appraisal

Studies were rated high quality (4/8, 50%) or moderate quality (4/8, 50%) based on the CASP systematic review checklist [52]. The CASP quality scores ranged from 18 to 25, with a mean of 22.1. The most common reason for low quality was a lack of reflexivity, where 50% (4/8) of the studies scored 0.

Table 2 outlines CASP scores for each study and their score for each domain. Domains are labeled 1 to 10 (1=clear aims, 2=appropriate qualitative methodology, 3=appropriate research design, 4=appropriate recruitment, 5=appropriate data collection, 6=considered reflexivity, 7=considered ethical issues, 8=rigor data analysis, 9=clear findings, and 10=value of research).

**Table 2:** Evaluation of the quality using the Critical Appraisal Skills Programme (CASP) systematic review checklist [52].

Study	CASP quality assessment score for each domain										
	Domain										CASP score^a^
	1	2	3	4	5	6	7	8	9	10	
Jarman et al [60], 2022	3	3	3	3	3	0	2	2	3	3	25
Juarascio et al [46], 2015	3	3	3	3	1	0	1	1	3	3	21
Linardon et al [69], 2022	3	3	0	1	2	2	1	1	2	3	18
McClay et al [66], 2013	3	3	3	3	3	0	2	3	3	3	26
Nitsch et al [72], 2016	3	3	3	0	2	2	1	3	2	2	21
Jafari [65], 2021	3	3	3	1	2	0	3	2	3	3	23
Yim et al [59], 2020	3	3	3	1	2	3	1	3	3	3	25
Yim et al [71], 2021	3	3	0	2	1	2	3	1	2	1	18

^a^The maximum overall score is 30. A total score <15 indicates the study is poor quality, 15 to 22.4 indicates moderate quality, and 22.5 to 30 indicates high quality. A score of 0 represents <25% of the criteria met, 1 being 25% to 49%, 2 being 50% to 74%, and 3 being 75% to 100%.

### Qualitative Analysis

#### Overview

The main findings of each study are outlined in Multimedia Appendix 3 [46,59,60,65,66,69,71,72].

A total of 7 metathemes and 13 subthemes were identified, as outlined in Table 3. Quotes provided are from the studies, as opposed to the studies’ participants.

**Table 3:** Metathemes and subthemes.

Metathemes	Subthemes
Appeal of digital interventions	Accessibility Anonymity Flexibility
Role of digital interventions in treatment	Stepping stone for therapy Alternative treatment
Value of support in treatment	Support from a professional Peer support
Communication at the right level	—^a^
Importance of engagement	—
Shaping knowledge to improve eating-disorder behaviors	Increased self-awareness and reflection Improvements in eating-disorder behaviors
Design of the digital intervention	Functions Media format User interface Customization

^a^No subthemes.

#### Theme 1: Appeal of Digital Interventions

Users across studies were initially drawn to using digital interventions because they were accessible [46,59,60,65,66,69,71] and allowed for anonymity [46,59,66,71,72].

##### Accessibility

Users were drawn to digital interventions as an accessible means of getting treatment. The intervention did not have long waitlists, unlike the traditional face-to-face interventions, and was low cost compared to other treatment options such as private psychotherapy [65,66,72].

Users found them accessible because they already owned a device to use the intervention on and no travel was required [60,72]:

[Digital interventions are] accessible for everyone because technology is omnipresent.

72

Digital interventions were also seen as accessible for those who were non-native English speakers as they provided features such as transcripts and audio recordings [69]. In addition, many users expressed optimism about digital interventions, recognizing their ability to address barriers they had personally encountered or were aware of in traditional treatment options [46,59,66,71].

##### Flexibility

Users identified flexibility to be a substantially appealing aspect of digital interventions. They perceived digital interventions as highly flexible and practical to use. This was attributed to the normalization of phone use across various situations and ease of access to the intervention through their mobile phone. Most users thought the intervention was compatible with their lifestyle and everyday routine [46,59,60,66,69,72]:

A key advantage...is the flexibility and freedom for the user to engage at any time.

60

##### Anonymity

Digital interventions were appealing because they offered a discrete method of help and the lack of face-to-face contact with a clinician made participants feel more comfortable [46,59,66,71,72]:

Self-help approach seemed like a desirable alternative to traditional face-to-face treatment, primarily because of the private, convenient, flexible, and anonymous nature.

66

Anonymity was appealing because some participants expressed shame and embarrassment about their ED symptoms or subtype, so preferred to keep treatment discrete from others [66,72]. Some participants saw technology and the internet as protection as it allowed them to remain anonymous. This made accessing treatment less daunting because they felt less vulnerable and more autonomous over their treatment [71,72].

#### Theme 2: The Role of Digital Interventions in Treatment

Across studies users saw digital interventions as less intensive and less effective compared to face-to-face treatment [59,60,69,71].

##### Stepping Stone for Therapy

Many users saw digital interventions as a means of early intervention or a prior step to getting traditional treatment—psychotherapy [59,60,65,69]. Therefore, users did not see digital interventions as a sufficient treatment to be used independently, but instead as a complementary tool to be used before or alongside psychotherapy:

Most regarded self-help as a prelude to getting “proper” treatment (“one tool in the toolbox”), rather than an alternative to traditional treatments.

71

##### Alternative Treatment

Many users had tried several treatment options for their ED, many of which were unsuccessful in helping them recover [46,65,66]. Some felt they had exhausted all their options for treatment and were failed by the health care system, therefore saw digital interventions as a tool users would seek out of desperation [59,66]:

Participants...had tried other approaches without success...and were open to learning a novel approach.

65

#### Theme 3: Value of Support

The digital interventions used in studies had varied levels of communication functions; some had direct contact with a psychologist, personal coach, or support worker [59,66,72]; some offered a community chat forum [59,72], and others did not offer either [46,60,65,69,71]. Nevertheless, users highly valued support from a professional [66,69,71] and peers [59,60,65,66,71].

##### Support From a Professional

Support from a professional helped users feel supported without being judged and kept them motivated by making them feel accountable [59,66]:

The support worker was a valued element...it was good to have someone there to help with their progress through the package without judgment: “...she has made a huge huge difference”

66

However, a minority felt pressured by the professional and disengaged as a result [50]. Users felt a reduced power imbalance with the professional compared to previous experiences with face-to-face therapy. However, this was at the cost of a reduced therapeutic alliance [71,72]. Due to this, some users still preferred face-to-face contact [72].

##### Peer Support

In the 2 studies that provided access to a discussion group [71,72], users highly valued peer support. One study [65] offered an intervention with neither support from peers nor a clinician, yet participants desired a discussion board to share mutual experiences and interact with others. One study, in which participants did not use an intervention but discussed their preferences hypothetically, found users valued peer support more than support from a professional [60]. Users thought that support from both peers and clinicians would increase motivation to use the intervention. Peer support could help users feel understood and reduce isolation and loneliness:

Being able to talk to people who are going through the same thing...is really important, because sometimes you just have questions that no one can answer even if they are a doctor or a therapist.

60

Similarly, a study that offered a discussion group [72] found increased motivation to continue treatment, as users thought having the option to communicate with other users reduced loneliness and increased their sense of connection and belonging. However, many were concerned with the potential harm of contact with others with an ED, as some users may share harmful thoughts or encourage each other’s ED:

Most participants (3/4, 75%) also acknowledged that this may be unhelpful or even dangerous if used inappropriately.

60

#### Theme 4: Communication at the Right Level

Many users suggested the intervention’s language had a key role in acceptability and motivation to continue use [46,60,72]. If they perceived insensitive language, they felt highly demotivated to continue using it [60,72].

Users disliked clinical language (eg, “binge eating episode”), acronyms (eg, “BED” and “CBT”), and formal language that made the intervention feel more serious and less enjoyable [46,60]. Users preferred informal and caring language, as this appeared more approachable and created a more pleasant experience [60]. However, there was an emphasis on a careful balance between professional and informal language [46]:

Wording and language used was an important issue as it was found to trigger negative emotions.

46

#### Theme 5: Importance of Engagement

Users highlighted facilitators that helped them initiate use and continue engagement. During initial use, users expressed the intervention’s interactivity and highly engaging content, and functions that kept them returning [65,69]. One user suggested a way to ensure engagement was to include frequent updates with new content and features. Users across studies explained that they were more likely to continue using if they were inputting data, specifically logging calories, moods, or binges [46,59,65,69]. If the intervention had predictable functions or familiar content, they were more likely to disengage and terminate use prematurely [46]:

User: “I would keep using the app if it continued to update. There has to be new features and new things about it.”

46

#### Theme 6: Shaping Knowledge to Improve ED Behaviors

##### Increased Self-Awareness and Reflection

Some users reported that the intervention resulted in positive changes in their ability and frequency of self-awareness, self-realization, and self-reflection. One study suggested that the intervention helped most users by promoting new knowledge and understanding of their ED [59].

Four studies reported that the majority of users noticed increased capability and frequency of self-reflection and self-awareness regarding their eating behaviors and beliefs about eating [46,59,65,72]. Users identified these positive impacts and attributed them to the intervention:

Participants described the overall positive impact of “holding off on an immediate desire” and learning to reflect on one’s feelings and actions.

65

##### Improvements In ED Behaviors

The majority of users from two studies were able to use their new skills and understanding of ED and apply them when they were about to eat [65,66]. In one study [65], most participants noticed that they were more mindful before and during eating. This led to more scheduled and mindful eating behaviors (ie, less eating when not hungry). These improved eating behaviors were attributed to the intervention:

Many participants described taking a pause to reflect before or during eating, which led to less snacking, less eating out of boredom, eating smaller quantities of food, and less night eating.

65

#### Theme 7: Design of the Digital Intervention

Users expressed several preferences relating to functions, media and format, and user interface. Within all studies except one, users heavily emphasized the importance of personalization and customization [46,59,60,65,69,71,72].

##### Functions

Users across studies found food logging functions to be controversial. Some desired the function as they felt it would aid self-awareness and found it convenient to be able to track multiple domains (eg, food, emotions, and binges) in one app [46,59,65,69]:

Nice if there was an area or section of it where you could log your food. Like what you’re eating so you could look back on it and see what you’re eating and how your habits are changing.

65

However, some users had previously been dependent on food logging, in the form of calorie counting, to feel a sense of control. These users shared the potential harm of a food logging function [65].

Some users highlighted that questionnaires and quizzes were perceived as insensitive as they felt they made light of a serious concern [65]. They preferred functions that aimed to manage negative emotions over functions and content that were more light-hearted and game-like.

##### Media Format

Those who used an intervention with text-heavy content found reading long texts to be a negative experience [65,72]. Those who experienced a greater variety of audio, images, and text content appreciated it, suggesting variation of media formats is preferred [46,69]. This extended to the format of data input, as users desired an option to input audio files and photos, particularly when logging moods or binges [60]:

Participants liked that there was a mixture of audio, images, and text-based content delivery formats, which is consistent with a recent quantitative study finding that people who would use e-health for ED symptoms stated a preference for such blended content delivery formats.

69

##### User Interface

Users had mixed preferences regarding design and appearance. Some preferred very simple and clear designs with minimal distractions [60,69,72], while others preferred more color and visually engaging material [69]:

Those that rated the visual design positively appreciated the simplicity of the layout...Several participants did not find the visual layout to be appealing, and suggested...more user-friendly images to better capture the attention of users.

69

Most users had positive experiences with navigating the interventions. This could have been improved by additional navigation functions (eg, a home button) and a more personalized user journey (eg, bookmarks and recent history) [59,65,72]. Many participants, in one study, explained this would reduce cognitive demand making it easier to maintain engagement and complete sessions.

Most users did not have technical difficulties, but for the few that did, this negatively impacted their experience. Technical difficulties made the intervention appear unreliable, reducing the motivation to continue use [65,66,72].

##### Customization to the Individual

Users highlighted that people with EDs have unique triggers, symptoms, and levels of understanding of their ED, which can heavily differ across individuals [46,59,65,66]. Users preferred more opportunities to customize the intervention’s functions and complexity of psychoeducation to meet their personal needs [59,65]. For example, users wanted to control the option to see more additional images if they had language needs or were non–native English speakers. Some users also wanted to have an option to turn on food logging functions, such as diaries of their daily food intake and calories, to feel a greater sense of control. Others wanted to turn off food-logging functions as they were perceived to be harmful and have the potential to trigger ED behaviors (eg, restriction or purging). Allowing users to customize functions and content would increase the relevance of content and increase motivation and commitment to use [60,65]:

The intervention was not a one size fits all and how the perceived usefulness and relevance were often dependent on participants’ demographic and clinical characteristics.

59

## Discussion

### Principal Findings

Most users across studies reported positive experiences of using digital interventions for their EDs. Digital interventions allowed for an accessible, flexible, and anonymous approach to treatment, which was appealing to users. They found them convenient, flexible, and easily integrated into their lifestyles. Digital interventions improved their understanding of EDs and increased self-reflection on their beliefs about eating and eating behaviors. This aided mindfulness during urges to binge, and when eating. Nevertheless, most users still perceived digital interventions for ED to be an early intervention or as a “last resort” after several unsuccessful treatment attempts. Some negative experiences were reported, which were attributed to technical difficulties, excessive text, feeling pressured by the support from a professional, and clinical, formal language. These highlight specific areas for improvement.

In terms of user preferences, there were mixed opinions on functions and user interface, highlighting the importance of customization. Food logging appeared to be the most controversial function. Some users found it convenient to be able to log moods and behaviors in one place, as they would otherwise have a separate food logging app, whereas others expressed concern about its potential harm and perpetuate their overconcern with food and weight. Opinions on user interface were also mixed as some preferred simple designs, while others preferred color and visually engaging material. The mix of opinions suggests digital interventions should have options for users to turn off functions they perceive as unhelpful and to customize the user interface (eg, color schemes, use of images, or potentially having a 2 user-interface options such as “simple” and “detailed”). In fact, most users expressed the importance of customization based on individual users having unique triggers, symptoms, and levels of understanding of their ED.

Most users expressed that the language and tone of content should feel approachable and be understandable to people with limited knowledge of ED (eg, no clinical terms or acronyms). Most found support from a professional and peers to be valuable, with users prioritizing peer support due to prevalent feelings of loneliness in their ED experience. However, a minority disagreed and perceived professional support in digital interventions to instill a power imbalance and a sense of pressure on users. Some users were also concerned about the potential harm of peer support, such as negative interactions or unhelpful information exchange.

Most users emphasized the importance of an intervention that met their language, lifestyle, and engagement needs, while recognizing their variation in ED symptoms and severity. An ideal intervention would allow users to tailor content, functions, appearance, navigation (eg, bookmarks and recent history), and format of content (eg, videos, text, and audio) to meet their own needs.

### Comparison With Prior Work

Our findings were generally consistent with prior work on digital interventions for EDs, yet provided some new insights.

Users’ perceptions on the benefits of professional and peer support were in line with a prior review suggesting that support from a professional was a source of motivation in self-help, which led to more satisfaction [47]. Similarly, support from peers or a professional was perceived to be an important motivator in encouraging engagement and adherence, consistent with previous literature.

Our findings were consistent with previous work on digital interventions for other mental health disorders. One review of 208 studies on digital interventions for mental health (depression, anxiety, and ED) and related issues (stress, mood, and anxiety) identified factors affecting user engagement [75]. Users were more likely to continue use if the content had perceived relevance, and if they had the option to personalize content, consistent with this study.

Another study on digital interventions for severe mental health problems (including bipolar and schizophrenia) suggested users found that digital interventions aided reflection and change, and preferred highly interactive interventions [76], consistent with our findings. Interestingly, data privacy concerns were more salient in the existing 2019 review and concerns were related to companies and clinicians accessing their data, whereas users in our review were more concerned with privacy from their friends and family [59,66] and less concerned about third-party companies [46].

We identified novel insights on user experiences, such as broader perceptions of digital interventions being an early intervention or “last resort” that were not mentioned in previous literature, as well as, user preferences, specifically what preferences were important for user engagement. New content and functions were important in maintaining engagement, so future interventions were recommended to occasionally update content and functions. We also found language used in digital interventions to be more important than previously suggested [21,47], as language had a key role in acceptability and motivation to continue use. Language was also an important factor in user experience, with language deemed insensitive or too formal substantially affecting users’ experiences negatively, which provided a novel insight. Further, the support from professionals elicited feelings of accountability, which was either motivating or disengaging for users—an insight not found in previous studies [21,47].

### Implications

This review supplements the literature on digital self-help interventions for EDs by exploring unguided self-help and by offering a qualitative exploration of the reasons behind low adherence. Previous reviews combined guided and unguided interventions, limiting their ability to draw firm conclusions about one or the other [47]. In addition, prior randomized controlled trials lacked the contextual understanding needed to explain low adherence due to their quantitative nature. Our qualitative review provides context as to why some people may not be engaging with digital interventions, and why there is low adherence [21].

Our findings can inform the design of digital interventions to better address the diverse needs and preferences of users with EDs. Key areas identified as important to most users include support from clinicians and peers, variation of media, and ways to ensure engagement, for instance, frequent updates and content for all knowledge levels.

The perceptions of digital interventions being an early intervention or “last resort” may suggest the need for flexible, adaptive approaches that can cater to users at different stages of their ED. For individuals viewing digital interventions as an early intervention, the design may prioritize easy accessibility, self-monitoring tools, and psychoeducation. For users who regard digital interventions as a “last resort,” it is important to include relevant emergency contact information and design elements that build trust and hope. These users may be skeptical about mental health interventions due to negative experiences with health care and mental health interventions. To address this, interventions may combine unguided and guided care, offering support and encouragement throughout the process while rebuilding users’ confidence in mental health support.

The findings from this review can help inform the design and development of digital interventions that address the existing “treatment gaps” for individuals with EDs. Digital interventions can be particularly beneficial for those with mild to moderate EDs who are attempting to access health care services but met with lengthy waitlists due to prioritization of severe EDs. Digital intervention can serve as the initial step in the stepped care model [77] to provide immediate access to mental health resources, psychological content, and supportive tools to help manage symptoms and distress.

### Strengths

Our methodology was inclusive because we searched published and unpublished studies from any country and published in any language. Our search strategy was developed with a librarian’s advice and expertise. This ensured that our search strategy was comprehensive and highly specific to our review questions. Two independent researchers completed screening, data extraction, and quality assessments, increasing this review’s reliability. Data extraction and quality appraisal were conducted independently by 2 researchers, reducing errors of inaccuracy [78].

Reflexivity was used to identify and understand researcher biases and their potential impact on our interpretation of the results. Our research team lacked lived experience of EDs, therefore, we addressed this by using patient and public involvement to identify misinterpretations or gaps in our analysis. We recruited 2 people with lived experience of ED and used their feedback to iteratively develop themes. This improved the validity of our analysis.

### Limitations

We aimed to be inclusive as we predicted limited relevant studies; however, this led to broad inclusion criteria and heterogeneity of study designs among included studies. Of the 8 included studies, 5 (63%) used study methods or interventions that were notably distinct. Of these, 2 (40%) did not require the use of a digital intervention [60,71], 1 (20%) used an intervention with a personal coach [72], 1 (20%) involved weekly communication and a online discussion board [44], and 1 (20%) study included weekly sessions with a support worker [66]. We included studies without a digital intervention [60,71] because we prioritized inclusivity, and they provided valuable insights on digital interventions more broadly and relative attitudes. However, because users were not speaking from experience, their preferences may be less accurate and may change after using an intervention, making our results less reliable. We included 3 studies that met our inclusion criteria for unguided interventions but still included some form of support, which may limit our review’s ability to make generalizations specifically about fully unguided interventions.

Our review lacked ethnic and demographic diversity. Our compiled group of users primarily consisted of White female university students from Western countries. Only 38% (3/8) of the studies reported sample ethnicity, of which most users were White [46,65,69]. Six studies used all-female samples [59,60,65,66,71,72]; all studies were conducted in high-income countries; and 63% (5/8) of the studies recruited from university populations [59,65,66,71,72]. This limits the generalizability of our findings to the broader ED population, where cultural contexts may have important influences on user experiences and preferences. Further research is required with participants from underrepresented groups (eg, men, older adults, nonuniversity students, ethnic minorities, non-Western countries, and economically deprived groups).

### Future Work

More research is needed to understand the role of digital interventions in care pathways. Future studies should explore their potential as the initial step in the stepped care model [77] and whether they can replace or complement face-to-face treatment. In addition, research should focus on making digital interventions adaptable to different stages of recovery, identifying which populations benefit most, and understanding user experiences across demographics.

Our findings emphasize the importance of support, whether from professionals or peers. Future work should compare user experiences of peer support versus professional support to determine what users find meaningful. It should also explore the right balance of support, as too much can create pressure and too little can reduce motivation, particularly across varying levels of ED severity.

Future interventions should empower users with more agency in logging functionality. For example, users could log meals with varying detail levels, scan barcodes, or select images. They could also log related information such as moods or recovery goal alignment using diverse formats such as emojis, free-text, images, or voice notes, enhancing convenience and personal expression.

Another potential pathway to increasing personalization may be through the use of artificial intelligence. One approach to this is by designing digital interventions to be “just-in time” adaptive interventions, by applying machine learning to food, mood, and binge inputted data to predict high-risk times for binging and restricting. For example, based on individual data, artificial intelligence may identify a high risk of binge when a user has had prolonged social media use after 10 PM and may send notifications for relevant content and exercises to prevent binges, purging, or restriction. Recent research proposes that the collection of data needed for machine learning, such as eating behaviors, physical activity, sleep and visual attention, can be collected by sensor technologies used in modern smartwatches and smartphones [79]. The current literature proposes machine learning for ED treatment is a cost-effective and rapid treatment option [80].

Our findings highlight the importance of a highly personalized user experience, raising questions about the role and impact of cocreation in this context. The existing literature recognizes the value of cocreation in developing needs-based services [81], including digital interventions for mental health [42], as it ensures interventions are aligned with the needs and preferences of the target population. Subsequently, digital interventions designed using cocreation have improved engagement, acceptability, user-friendliness, and cultural sensitivity [81-83].

However, there is limited understanding on the direct effects of cocreation on improving digital interventions. Future work may use cocreation to involve end users in all stages of design and intervention development, a practice that is a core principle of cocreation but rarely practiced [84,85] and explore the impact of cocreation on increasing personalization and improving appropriateness of language.

### Conclusions

Users reported positive experiences with digital interventions, valuing their flexibility, anonymity, and convenience. These tools improved their understanding of EDs, facilitated self-reflection, and promoted coping skills such as mindfulness during time of distress. Both peer and professional support were appreciated for building trust and empathy, enhancing user engagement.

Opinions varied across a range of features, such as food logging and user interface design. This shows the importance of personalization, allowing for customization of features based on individual preferences and triggers, which could significantly improve user engagement. However, it is crucial to ensure that digital interventions use components that are effective in ED treatment and are delivered in a way that is acceptable to the end user.

These insights provide clear direction for future codesign and development of digital interventions. Further involvement of end users from early research through to final testing will allow developers to create more engaging, personalized, and effective tools that better meet user needs, ultimately reducing dropout rates and closing the treatment gap for those with mild to moderate EDs.

## Appendix

Multimedia Appendix 1 PRISMA (Preferred Reporting Items for Systematic Reviews and Meta-Analyses) checklist.

Multimedia Appendix 2 Full search strategy.

Multimedia Appendix 3 Main findings for individual studies.

## Abbreviations

CASP: Critical Appraisal Skills Programme
CBT: cognitive behavioral therapy
ED: eating disorder
PRISMA: Preferred Reporting Items for Systematic Reviews and Meta-Analyses

## References

[1] Galmiche M, Déchelotte P, Lambert G, Tavolacci MP (2019). Prevalence of eating disorders over the 2000-2018 period: a systematic literature review. Am J Clin Nutr.

[2] Ayton A, Viljoen D, Ryan S, Ibrahim A, Ford D (2022). Risk, demand, capacity and outcomes in adult specialist eating disorder services in South-East of England before and since COVID-19. BJPsych Bull.

[3] Kazdin AE, Fitzsimmons-Craft EE, Wilfley DE (2017). Addressing critical gaps in the treatment of eating disorders. Int J Eat Disord.

[4] Hansen SJ, Stephan A, Menkes DB (2021). The impact of COVID-19 on eating disorder referrals and admissions in Waikato, New Zealand. J Eat Disord.

[5] Vuillier L, May L, Greville-Harris M, Surman R, Moseley RL (2021). The impact of the COVID-19 pandemic on individuals with eating disorders: the role of emotion regulation and exploration of online treatment experiences. J Eat Disord.

[6] Fursland A, Erceg-Hurn DM, Byrne SM, McEvoy PM (2018). A single session assessment and psychoeducational intervention for eating disorders: impact on treatment waitlists and eating disorder symptoms. Int J Eat Disord.

[7] Austin A, Flynn M, Richards K, Hodsoll J, Duarte TA, Robinson P, Kelly J, Schmidt U (2021). Duration of untreated eating disorder and relationship to outcomes: a systematic review of the literature. Eur Eat Disord Rev.

[8] Reyes-Rodríguez ML, Ramírez J, Davis K, Patrice K, Bulik CM (2013). Exploring barriers and facilitators in eating disorders treatment among Latinas in the United States. J Lat Psychol.

[9] Innes NT, Clough BA, Casey LM (2017). Assessing treatment barriers in eating disorders: a systematic review. Eat Disord.

[10] Hildebrandt T, Michaeledes A, Mayhew M, Greif R, Sysko R, Toro-Ramos T, DeBar L (2020). Randomized Controlled Trial Comparing Health Coach-Delivered Smartphone-Guided Self-Help With Standard Care for Adults With Binge Eating. American Journal of Psychiatry.

[11] Makowski AC, Mnich EE, Angermeyer MC, Löwe B, von dem Knesebeck O (2015). Sex differences in attitudes towards females with eating disorders. Eat Behav.

[12] Räisänen U, Hunt K (2014). The role of gendered constructions of eating disorders in delayed help-seeking in men: a qualitative interview study. BMJ Open.

[13] Hamilton A, Mitchison D, Basten C, Byrne S, Goldstein M, Hay P, Heruc G, Thornton C, Touyz S (2022). Understanding treatment delay: perceived barriers preventing treatment-seeking for eating disorders. Aust N Z J Psychiatry.

[14] McNicholas F, O'Connor C, O'Hara L, McNamara N (2016). Stigma and treatment of eating disorders in Ireland: healthcare professionals' knowledge and attitudes. Ir J Psychol Med.

[15] Wacker EC (2018). Barriers and facilitators to seeking treatment for subclinical eating disorders: the importance of supportive relationships. J Fam Psychother.

[16] Linardon J, Cuijpers P, Carlbring P, Messer M, Fuller-Tyszkiewicz M (2019). The efficacy of app-supported smartphone interventions for mental health problems: a meta-analysis of randomized controlled trials. World Psychiatry.

[17] Eating disorders: recognition and treatment. National Institute for Health and Care Excellence.

[18] Tregarthen JP, Lock J, Darcy AM (2015). Development of a smartphone application for eating disorder self-monitoring. Int J Eat Disord.

[19] Lindgreen P, Clausen L, Lomborg K (2018). Clinicians' perspective on an app for patient self-monitoring in eating disorder treatment. Int J Eat Disord.

[20] Vollert B, Beintner I, Musiat P, Gordon G, Görlich D, Nacke B, Schmidt-Hantke J, Potterton R, Spencer L, Grant N, Schmidt U, Jacobi C (2019). Using internet-based self-help to bridge waiting time for face-to-face outpatient treatment for Bulimia Nervosa, Binge Eating Disorder and related disorders: study protocol of a randomized controlled trial. Internet Interv.

[21] Linardon J, Shatte A, Messer M, Firth J, Fuller-Tyszkiewicz M (2020). E-mental health interventions for the treatment and prevention of eating disorders: an updated systematic review and meta-analysis. J Consult Clin Psychol.

[22] Barakat S, Maguire S, Smith KE, Mason TB, Crosby RD, Touyz S (2019). Evaluating the role of digital intervention design in treatment outcomes and adherence to eTherapy programs for eating disorders: a systematic review and meta-analysis. Int J Eat Disord.

[23] Melioli T, Bauer S, Franko DL, Moessner M, Ozer F, Chabrol H, Rodgers RF (2016). Reducing eating disorder symptoms and risk factors using the internet: a meta-analytic review. Int J Eat Disord.

[24] Ter Huurne ED, Postel MG, de Haan HA, van der Palen J, DeJong CA (2017). Treatment dropout in web-based cognitive behavioral therapy for patients with eating disorders. Psychiatry Res.

[25] Aardoom JJ, Dingemans AE, Spinhoven P, Van Furth EF (2013). Treating eating disorders over the internet: A systematic review and future research directions. The International journal of eating disorders.

[26] Melville KM, Casey LM, Kavanagh DJ (2010). Dropout from Internet-based treatment for psychological disorders. Br J Clin Psychol.

[27] Kelders SM, Kok RN, Ossebaard HC, Van Gemert-Pijnen JE (2012). Persuasive system design does matter: a systematic review of adherence to web-based interventions. J Med Internet Res.

[28] Schlegl S, Bürger C, Schmidt L, Herbst N, Voderholzer U (2015). The potential of technology-based psychological interventions for anorexia and bulimia nervosa: a systematic review and recommendations for future research. J Med Internet Res.

[29] Linardon J, Hindle A, Brennan L (2018). Dropout from cognitive-behavioral therapy for eating disorders: A meta-analysis of randomized, controlled trials. The International journal of eating disorders.

[30] Boucher EM, Ward HE, Mounts AC, Parks AC (2021). Engagement in digital mental health interventions: can monetary incentives help?. Front Psychol.

[31] Graves TA, Tabri N, Thompson-Brenner H, Franko DL, Eddy KT, Bourion-Bedes S, Brown A, Constantino MJ, Flückiger C, Forsberg S, Isserlin L, Couturier J, Paulson Karlsson G, Mander J, Teufel M, Mitchell JE, Crosby RD, Prestano C, Satir DA, Simpson S, Sly R, Lacey JH, Stiles-Shields C, Tasca GA, Waller G, Zaitsoff SL, Rienecke R, Le Grange D, Thomas JJ (2017). A meta-analysis of the relation between therapeutic alliance and treatment outcome in eating disorders. Int J Eat Disord.

[32] Saekow J, Jones M, Gibbs E, Jacobi C, Fitzsimmons-Craft EE, Wilfley D, Barr Taylor C (2015). StudentBodies-eating disorders: a randomized controlled trial of a coached online intervention for subclinical eating disorders. Internet Interv.

[33] Jiotsa B, Naccache B, Duval M, Rocher B, Grall-Bronnec M (2021). Social media use and body image disorders: association between frequency of comparing one's own physical appearance to that of people being followed on social media and body dissatisfaction and drive for thinness. Int J Environ Res Public Health.

[34] Kaveladze BT, Wasil AR, Bunyi JB, Ramirez V, Schueller SM (2022). User Experience, Engagement, and Popularity in Mental Health Apps: Secondary Analysis of App Analytics and Expert App Reviews. JMIR Human Factors.

[35] Chen AT, Wu S, Tomasino KN, Lattie EG, Mohr DC (2019). A multi-faceted approach to characterizing user behavior and experience in a digital mental health intervention. J Biomed Inform.

[36] Naccache B, Mesquida L, Raynaud JP, Revet A (2021). Smartphone application for adolescents with anorexia nervosa: an initial acceptability and user experience evaluation. BMC Psychiatry.

[37] Anastasiadou D, Folkvord F, Serrano-Troncoso E, Lupiañez-Villanueva F (2019). Mobile health adoption in mental health: user experience of a mobile health app for patients with an eating disorder. JMIR Mhealth Uhealth.

[38] Anastasiadou D, Folkvord F, Lupiañez-Villanueva F (2018). A systematic review of mHealth interventions for the support of eating disorders. Eur Eat Disorders Rev.

[39] Torous J, Nicholas J, Larsen ME, Firth J, Christensen H (2018). Clinical review of user engagement with mental health smartphone apps: evidence, theory and improvements. Evid Based Ment Health.

[40] Linardon J, Messer M, Lee S, Rosato J (2021). Perspectives of e-health interventions for treating and preventing eating disorders: descriptive study of perceived advantages and barriers, help-seeking intentions, and preferred functionality. Eat Weight Disord.

[41] Keeler JL, Peters-Gill G, Treasure J, Himmerich H, Tchanturia K, Cardi V (2022). Difficulties in retrieving specific details of autobiographical memories and imagining positive future events in individuals with acute but not remitted anorexia nervosa. J Eat Disord.

[42] Graham AK, Wildes JE, Reddy M, Munson SA, Barr Taylor C, Mohr DC (2019). User-centered design for technology-enabled services for eating disorders. Int J Eat Disord.

[43] Keeler JL, Chami R, Cardi V, Hodsoll J, Bonin E, MacDonald P, Lawrence N (2022). App-based food-specific inhibitory control training as an adjunct to treatment as usual in binge-type eating disorders: A feasibility trial. Science Direct.

[44] The growing value of digital health. IQVIA Institute.

[45] Apps and connected devices in healthcare. IQVIA.

[46] Juarascio AS, Goldstein SP, Manasse SM, Forman EM, Butryn ML (2015). Perceptions of the feasibility and acceptability of a smartphone application for the treatment of binge eating disorders: qualitative feedback from a user population and clinicians. Int J Med Inform.

[47] Yim SH, Schmidt U (2019). Experiences of computer-based and conventional self-help interventions for eating disorders: a systematic review and meta-synthesis of qualitative research. Int J Eat Disord.

[48] Moher D, Liberati A, Tetzlaff J, Altman DG, PRISMA Group (2010). Preferred reporting items for systematic reviews and meta-analyses: the PRISMA statement. Int J Surg.

[49] Loucas C, Pennant M, Whittington C, Naqvi S, Sealey C, Stockton S, Kelvin R, Fonagy P, Kendall T (2014). G130 E-therapies for mental health problems in children and young people: a systematic review and focus group investigation. Arch Dis Child.

[50] Endnote. Clarivate.

[51] The world's #1 systematic review tool. Covidence.

[52] CASP checklists. Critical Appraisal Skills Programme.

[53] Cesario S, Morin K, Santa-Donato A (2002). Evaluating the level of evidence of qualitative research. J Obstet Gynecol Neonatal Nurs.

[54] Home page. NVivo.

[55] Thomas J, Harden A (2008). Methods for the thematic synthesis of qualitative research in systematic reviews. BMC Med Res Methodol.

[56] Nicholson E, Murphy T, Larkin P, Normand C, Guerin S (2016). Protocol for a thematic synthesis to identify key themes and messages from a palliative care research network. BMC Res Notes.

[57] Finlayson K, Crossland N, Bonet M, Downe S (2020). What matters to women in the postnatal period: a meta-synthesis of qualitative studies. PLoS One.

[58] Burnham J, Krause IB (2022). Developments in social GRRRAAACCEEESSS: visible–invisible and voiced–unvoiced 1. Culture and Reflexivity in Systemic Psychotherapy: Mutual Perspectives.

[59] Yim SH, Bailey E, Gordon G, Grant N, Musiat P, Schmidt U (2020). Exploring participants' experiences of a web-based program for bulimia and binge eating disorder: qualitative study. J Med Internet Res.

[60] Jarman HK, McLean SA, Rodgers R, Fuller-Tyszkiewicz M, Paxton S, O'Gorman B, Harris E, Shatte A, Bishop K, Baumann T, Mahoney D, Daugelat M, Yager Z (2022). Informing mHealth and web-based eating disorder interventions: combining lived experience perspectives with design thinking approaches. JMIR Form Res.

[61] Jacobi C, Hayward C, de Zwaan M, Kraemer HC, Agras WS (2004). Coming to terms with risk factors for eating disorders: application of risk terminology and suggestions for a general taxonomy. Psychol Bull.

[62] Data for high income, United States. The World Bank Group.

[63] Data for high income, United Kingdom. The World Bank Group.

[64] Data for high income, Australia. The World Bank Group.

[65] Jafari N (2021). Assessing the feasibility, acceptability, preliminary efficacy, and anticipated clinical practice implementation of a mindful eating smartphone application: a mixed methods analysis among undergraduate women with binge eating and clinical experts. The University of North Carolina at Charlotte.

[66] McClay CA, Waters L, McHale C, Schmidt U, Williams C (2013). Online cognitive behavioral therapy for bulimic type disorders, delivered in the community by a nonclinician: qualitative study. J Med Internet Res.

[67] McClay CA, Waters L, Schmidt U, Williams C (2016). A survey of attitudes towards computerized self-help for eating disorders within a community-based sample. Behav Cogn Psychother.

[68] Fairburn CG, Beglin SJ (2008). Cognitive Behavior Therapy and Eating Disorders.

[69] Linardon J, King T, Shatte A, Fuller-Tyszkiewicz M (2022). Usability evaluation of a cognitive-behavioral app-based intervention for binge eating and related psychopathology: a qualitative study. Behav Modif.

[70] Linardon J, Rosato J, Messer M (2020). Break Binge Eating: reach, engagement, and user profile of an internet-based psychoeducational and self-help platform for eating disorders. Int J Eat Disord.

[71] Yim SH, Spencer L, Gordon G, Allen KL, Musiat P, Schmidt U (2021). Views on online self-help programmes from people with eating disorders and their carers in UK. Eur J Public Health.

[72] Nitsch M, Dimopoulos CN, Flaschberger E, Saffran K, Kruger JF, Garlock L, Wilfley DE, Taylor CB, Jones M (2016). A guided online and mobile self-help program for individuals with eating disorders: an iterative engagement and usability study. J Med Internet Res.

[73] Jones M, Kass AE, Trockel M, Glass AI, Wilfley DE, Taylor CB (2014). A population-wide screening and tailored intervention platform for eating disorders on college campuses: the healthy body image program. J Am Coll Health.

[74] Morgan JF, Reid F, Lacey JH (1999). The SCOFF questionnaire: assessment of a new screening tool for eating disorders. BMJ.

[75] Borghouts J, Eikey E, Mark G, De Leon C, Schueller SM, Schneider M, Stadnick N, Zheng K, Mukamel D, Sorkin DH (2021). Barriers to and facilitators of user engagement with digital mental health interventions: systematic review. J Med Internet Res.

[76] Berry N, Lobban F, Bucci S (2019). A qualitative exploration of service user views about using digital health interventions for self-management in severe mental health problems. BMC Psychiatry.

[77] Kaltenthaler E, Shackley P, Stevens K, Beverley C, Parry G, Chilcott J (2002). A systematic review and economic evaluation of computerised cognitive behaviour therapy for depression and anxiety. Health Technol Assess.

[78] Buscemi N, Hartling L, Vandermeer B, Tjosvold L, Klassen TP (2006). Single data extraction generated more errors than double data extraction in systematic reviews. J Clin Epidemiol.

[79] Presseller EK, Patarinski AG, Fan SC, Lampe EW, Juarascio AS (2022). Sensor technology in eating disorders research: a systematic review. Int J Eat Disord.

[80] Fardouly J, Crosby RD, Sukunesan S (2022). Potential benefits and limitations of machine learning in the field of eating disorders: current research and future directions. J Eat Disord.

[81] Konttila J, Korkiakoski V, Kurikka J, Pääkkönen J, Kyngäs H (2021). Co-creation : an approach to developing digitalized mental healthcare. Psychiatr Fennica.

[82] Ricciardi W, Pita Barros P, Bourek A, Brouwer W, Kelsey T, Lehtonen L, Expert Panel on Effective Ways of Investing in Health (EXPH) (2019). How to govern the digital transformation of health services. Eur J Public Health.

[83] Porche MV, Folk JB, Tolou-Shams M, Fortuna LR (2022). Researchers' perspectives on digital mental health intervention co-design with marginalized community stakeholder youth and families. Front Psychiatry.

[84] Chauhan A, Walton M, Manias E, Walpola RL, Seale H, Latanik M, Leone D, Mears S, Harrison R (2020). The safety of health care for ethnic minority patients: a systematic review. Int J Equity Health.

[85] Brotherdale R, Berry K, Branitsky A, Bucci S (2024). Co-producing digital mental health interventions: a systematic review. Digit Health.

