# Supplementary material for: User Experiences of and Preferences for Self-Guided Digital Interventions for the Treatment of Mild to Moderate Eating Disorders: Systematic Review and Metasynthesis
Source: JMIR Ment Health. 2025 Jan 3;12:e57795. doi: 10.2196/57795 (PMC11748441; doi:10.2196/57795)
Supplement: Multimedia Appendix 2 [file mental_v12i1e57795_app2.docx]

**Multimedia Appendix 2**

**Full Search Strategy**

***Medline (Ovid)***

**Database:**
Ovid MEDLINE(R) ALL

| **#** | **Query** |
| --- | --- |
| 1 | exp "Feeding and Eating Disorders"/ |
| 2 | anorexi*.ti,ab. |
| 3 | (bing* adj2 eat*).ti,ab. |
| 4 | bulimi*.ti,ab. |
| 5 | EDNOS.ti,ab. |
| 6 | "eating disorder not otherwise specified".ti,ab. |
| 7 | OSFED.ti,ab. |
| 8 | "other specified feeding or eating disorder*".ti,ab. |
| 9 | appetite disorder*.ti,ab. |
| 10 | feeding disorder*.ti,ab. |
| 11 | eating disorder*.ti,ab. |
| 12 | (compulsiv* adj2 (eat* or vomit*)).ti,ab. |
| 13 | overeat*.ti,ab. |
| 14 | over-eat*.ti,ab. |
| 15 | restricted eating.ti,ab. |
| 16 | induc* vomit.ti,ab. |
| 17 | 1 or 2 or 3 or 4 or 5 or 6 or 7 or 8 or 9 or 10 or 11 or 12 or 13 or 14 or 15 or 16 |
| 18 | Computers, Handheld/ |
| 19 | Cell Phone/ or Smartphone/ |
| 20 | (Tablet* and (device* or computer*)).tw. |
| 21 | Web Browser/ |
| 22 | Mobile Applications/ |
| 23 | Computer program/ |
| 24 | ((Computer* or internet*or digital* or online*) and deliver*).tw. |
| 25 | ((mobile* or cell* or smart*) and phone*).tw. |
| 26 | ((sms or mms) and message*).tw. |
| 27 | (ios or android*).tw. |
| 28 | (ipad* or iphone* or ipod*).tw. |
| 29 | computer assisted therapy/ |
| 30 | ((online or web*) and (education* or train*)).tw. |
| 31 | (e health or ehealth or mhealth or m health or telehealth*or etherap* or e therap* or e mental* or emental).tw. |
| 32 | 18 or 19 or 20 or 21 or 22 or 23 or 24 or 25 or 26 or 27 or 28 or 29 or 30 or 31 |
| 33 | "Attitude to Computers"/ |
| 34 | exp "Treatment Adherence and Compliance"/ |
| 35 | (experience* or prefer* or thought* or perception* or opinion* or suggestion* or attitude*).tw. |
| 36 | exp Qualitative Research/ |
| 37 | user*.tw. |
| 38 | patient*.tw. |
| 39 | accept*.tw. |
| 40 | (adher* or nonadher* or non-adher*).tw. |
| 41 | (complian* or noncomplian* or non complian*).tw. |
| 42 | (refusal or refuse*).tw. |
| 43 | 33 or 34 or 35 or 36 or 37 or 38 or 39 or 40 or 41 or 42 |
| 44 | 17 and 32 and 43 |
| 45 | limit 44 to yr="2013 -Current" |

***Embase (Ovid)***

**Database:**
Embase

| **#** | **Query** |
| --- | --- |
| 1 | eating disorder/ or anorexia nervosa/ or avoidant restrictive food intake disorder/ or binge eating disorder/ or bulimia/ or emotional eating/ or food addiction/ or food aversion/ or food refusal/ or orthorexia/ or pica/ or purging disorder/ |
| 2 | anorexi*.ti,ab. |
| 3 | (bing* adj2 eat*).ti,ab. |
| 4 | bulimi*.ti,ab. |
| 5 | EDNOS.ti,ab. |
| 6 | "eating disorder not otherwise specified".ti,ab. |
| 7 | OSFED.ti,ab. |
| 8 | "other specified feeding or eating disorder*".ti,ab. |
| 9 | appetite disorder*.ti,ab. |
| 10 | feeding disorder*.ti,ab. |
| 11 | eating disorder*.ti,ab. |
| 12 | (compulsiv* adj2 (eat* or vomit*)).ti,ab. |
| 13 | overeat*.ti,ab. |
| 14 | over-eat*.ti,ab. |
| 15 | restricted eating.ti,ab. |
| 16 | induc* vomit.ti,ab. |
| 17 | 1 or 2 or 3 or 4 or 5 or 6 or 7 or 8 or 9 or 10 or 11 or 12 or 13 or 14 or 15 or 16 |
| 18 | exp personal computer/ |
| 19 | personal digital assistant/ |
| 20 | computer assisted therapy/ |
| 21 | desktop computer/ |
| 22 | exp tablet computer/ |
| 23 | exp mobile phone/ or smartphone/ |
| 24 | mobile phone/ |
| 25 | exp mobile phone/ |
| 26 | smartphone/ |
| 27 | exp tablet computer/ |
| 28 | exp web browser/ |
| 29 | exp mobile application/ |
| 30 | ((Computer* or internet*or digital* or online*) and deliver*).tw. |
| 31 | ((mobile* or cell* or smart*) and phone*).tw. |
| 32 | ((sms or mms) and message*).tw. |
| 33 | (ios or android*).tw. |
| 34 | (ipad* or iphone* or ipod*).tw. |
| 35 | ((online or web*) and (education* or train*)).tw. |
| 36 | (e health or ehealth or mhealth or m health or telehealth*or etherap* or e therap* or e mental* or emental).tw. |
| 37 | exp telehealth/ or health care delivery/ or telecommunication/ |
| 38 | 18 or 19 or 20 or 21 or 22 or 23 or 24 or 25 or 26 or 27 or 28 or 29 or 30 or 31 or 32 or 33 or 34 or 35 or 36 or 37 |
| 39 | exp attitude to computers/ |
| 40 | attitude to health/ |
| 41 | exp patient compliance/ |
| 42 | patient attitude/ or attitude/ or health care quality/ or patient attendance/ or patient compliance/ or patient dropout/ or patient engagement/ or patient participation/ or patient preference/ or patient satisfaction/ or refusal to participate/ or treatment interruption/ or treatment refusal/ |
| 43 | (experience* or prefer* or thought* or perception* or opinion* or suggestion* or attitude*).tw. |
| 44 | exp qualitative research/ |
| 45 | user*.tw. |
| 46 | ((user* or patient*) adj4 (experience* or prefer* or thought* or perception* or opinion* or suggestion* or attitude*)).tw. |
| 47 | accept*.tw. |
| 48 | (adher* or nonadher* or non-adher*).tw. |
| 49 | (complian* or noncomplian* or non complian*).tw. |
| 50 | (refusal or refuse*).tw. |
| 51 | 39 or 40 or 41 or 42 or 43 or 44 or 45 or 46 or 47 or 48 or 49 or 50 |
| 52 | 17 and 38 and 51 |
| 53 | limit 52 to yr="2013 -Current" |

***PsycINFO (OVID)***

Database:
APA PsycInfo

| # | Query |
| --- | --- |
| 1 | exp Eating Disorders/ |
| 2 | anorexi*.ti,ab. |
| 3 | (bing* adj2 eat*).ti,ab. |
| 4 | bulimi*.ti,ab. |
| 5 | EDNOS.ti,ab. |
| 6 | "eating disorder not otherwise specified".ti,ab. |
| 7 | OSFED.ti,ab. |
| 8 | "other specified feeding or eating disorder*".ti,ab. |
| 9 | appetite disorder*.ti,ab. |
| 10 | feeding disorder*.ti,ab. |
| 11 | eating disorder*.ti,ab. |
| 12 | (compulsiv* adj2 (eat* or vomit*)).ti,ab. |
| 13 | overeat*.ti,ab. |
| 14 | over-eat*.ti,ab. |
| 15 | restricted eating.ti,ab. |
| 16 | induc* vomit.ti,ab. |
| 17 | 1 or 2 or 3 or 4 or 5 or 6 or 7 or 8 or 9 or 10 or 11 or 12 or 13 or 14 or 15 or 16 |
| 18 | exp Computers/ |
| 19 | Computer Software/ |
| 20 | Personal Computers/ |
| 21 | Computer Based Training/ |
| 22 | Computer Applications/ |
| 23 | Computer Assisted Therapy/ |
| 24 | exp Electronic Learning/ or Mobile Applications/ |
| 25 | exp Mobile Devices/ |
| 26 | ((Computer* or internet*or digital* or online*) and deliver*).tw. |
| 27 | ((mobile* or cell* or smart*) and phone*).tw. |
| 28 | ((sms or mms) and message*).tw. |
| 29 | (ios or android*).tw. |
| 30 | (ipad* or iphone* or ipod*).tw. |
| 31 | ((online or web*) and (education* or train*)).tw. |
| 32 | (e health or ehealth or mhealth or m health or telehealth*or etherap* or e therap* or e mental* or emental).tw. |
| 33 | 18 or 19 or 20 or 21 or 22 or 23 or 24 or 25 or 26 or 27 or 28 or 29 or 30 or 31 or 32 |
| 34 | "Attitude to Computers".tw. |
| 35 | Treatment Dropouts/ or Treatment Barriers/ |
| 36 | exp Patient Satisfaction/ |
| 37 | Patient Dropouts/ or Patient Participation/ or Outpatient Treatment/ or Patient Attitudes/ or Patient Adherence/ |
| 38 | Patient Dropouts/ or Patient Participation/ or Patient Attitudes/ or Patient Adherence/ |
| 39 | Outpatient Treatment/ |
| 40 | Outpatient Commitment/ |
| 41 | Patient Education/ |
| 42 | (experience* or prefer* or thought* or perception* or opinion* or suggestion* or attitude*).tw. |
| 43 | exp Qualitative Research/ |
| 44 | user*.tw. |
| 45 | ((user* or patient*) adj4 (experience* or prefer* or thought* or perception* or opinion* or suggestion* or attitude*)).tw. |
| 46 | accept*.tw. |
| 47 | (adher* or nonadher* or non-adher*).tw. |
| 48 | (complian* or noncomplian* or non complian*).tw. |
| 49 | (refusal or refuse*).tw. |
| 50 | 34 or 35 or 36 or 37 or 38 or 39 or 40 or 41 or 42 or 43 or 44 or 45 or 46 or 47 or 48 or 49 |
| 51 | 17 and 33 and 50 |
| 52 | limit 51 to yr="2013 -Current" |

***Web of Science (Core Collection)***

<https://www.webofscience.com/wos/woscc/summary/f22731e6-4d9b-49fa-8088-d094f3543655-7fb95766/relevance/1>

(topic):

“eating disorder*" or anorexi* or "bing* eat*" or bulimi* or OSFED* or "Other specified feeding or eating disorder*" or "appetite disorder*" or "feeding disorder*" OR (disorder* NEAR/1 eat*) or (bing* NEAR/1 eat*) or (Compulsiv* NEAR/1 (eat* or vomit*)) OR overeat* or "over-eat*" OR "restrict* eat*" OR "induce* vomit*"

AND

(topic):

computer* or PC or “personal computer*” or ((mobile* or cell* or smart*) and phone*)

or (Tablet* and (device* or computer*)) or “web browser*” or website* or “mobile app*” or “computer program*” or “computer* app*” or “ smartphone app*” or ios or android or ipad* or iphone* or ipod* or ((sms or mms) and message*) or “computer assisted therapy*” or ((computer* or internet* or digital* or online*) and deliver*) or ((online or web*) and (education* or train*)) or “e health” or ehealth or mhealth or “m health” or telehealth* or etherap* or “e therap*” or “e mental*” or emental

AND

(topic):

((user* or patient*) NEAR/4 (experience* or prefer* or thought* or perception* or opinion* or suggest* or attitude*)) or accept* or adher* or nonadher* or “non-adher*” or complian* or noncomplian* or “non complian*” or refusal or refuse* or qualitative or dropout* or “treatment barrier*” or satisf* or user* or refusal or refuse*

Limit from 2013-01-01 to 2024-07-13

***CINAHL (EBSCOhost)***

https://web.p.ebscohost.com/ehost/resultsadvanced?vid=67&sid=705a3b85-d5f9-49be-8e2f-69c28a47e3f3%40redis&bquery=AB+((treatment+N2+(adher*+or+compli*+or+nonadher*+or+non-adher*+or+noncomplian+or+%e2%80%9cnon-complian%e2%80%9d+or+refuse*+or+refusal))+or+qualitative*+or+((user*+or+patient*)+N4+(experience*+or+prefer*+or+thought*+or+perception*+or+opinion*+or+suggestion*+or+attitude*))+or+acceptan*)&bdata=JkF1dGhUeXBlPWlwLHNoaWImZGI9amxoJnR5cGU9MSZzZWFyY2hNb2RlPVN0YW5kYXJkJnNpdGU9ZWhvc3QtbGl2ZSZzY29wZT1zaXRl

| S38 | S34 AND S35 AND S36 | Limiters - Publication Year: 2013-2024  Expanders - Apply equivalent subjects  Search modes - Boolean/Phrase |  |
| --- | --- | --- | --- |
| 527 results   | S37 | S34 AND S35 AND S36 |  |
|  | S36 | S24 OR S27 OR S30 or S33 |  |
|  | S35 | S12 OR S15 OR S18 OR S21 |  |
|  | S34 | S3 OR S6 OR S9 |  |
|  | S33 | S31 OR S32 |  |
|  | S32 | AB ((treatment N2 (adher* or compli* or nonadher* or non-adher* or noncomplian or “non-complian” or refuse* or refusal)) or qualitative* or ((user* or patient*) N4 (experience* or prefer* or thought* or perception* or opinion* or suggestion* or attitude*)) or acceptan*) |  |
|  | S31 | TI ((treatment N2 (adher* or compli* or nonadher* or non-adher* or noncomplian or “non-complian” or refuse* or refusal)) or qualitative* or ((user* or patient*) N4 (experience* or prefer* or thought* or perception* or opinion* or suggestion* or attitude*)) or acceptan*) |  |
|  | S30 | S28 OR S29 |  |
|  | S29 | AB ("Patient Attitudes") |  |
|  | S28 | TI ("Patient Attitudes") |  |
|  | S27 | S25 OR S26 |  |
|  | S26 | AB ((MH "Treatment Withdrawal") OR (MH "Treatment Duration") OR (MH "Treatment Termination") OR (MH "Attitude to Medical Treatment") OR (MH "Treatment Refusal") ) |  |
|  | S25 | TI ((MH "Treatment Withdrawal") OR (MH "Treatment Duration") OR (MH "Treatment Termination") OR (MH "Attitude to Medical Treatment") OR (MH "Treatment Refusal") ) |  |
|  | S24 | S22 OR S23 |  |
|  | S23 | TI ((MH "Patient Satisfaction+") ) |  |
|  | S22 | TI ((MH "Patient Satisfaction+") ) |  |
|  | S21 | S19 OR S20 |  |
|  | S20 | AB ((MH "Web Browsers") OR (MH "World Wide Web") OR (MH "World Wide Web Applications") OR (MH "Internet-Based Intervention") ) |  |
|  | S19 | TI ((MH "Web Browsers") OR (MH "World Wide Web") OR (MH "World Wide Web Applications") OR (MH "Internet-Based Intervention") ) |  |
|  | S18 | S16 OR S17 |  |
|  | S17 | AB (computer* or PC or “personal computer*” or “cell phone*” or “smartphone” or “smart phone*” or “mobile phone*” or (Tablet* and (device* or computer*)) or “web browser*” or website* or “mobile app”* or “computer program*” or “smartphone app*” or ios or android or ipad* or iphone* or ipod* or “computer assisted therapy*” or computer* or internet* or digital* or online* or deliver* or ((online or web*) and (education* or train*)) or “e health” or ehealth or mhealth or “m health” or telehealth or [...](javascript:showHistoryTerm('ctl00_ctl00_FindField_FindField_historyControl_HistoryRepeater_ctl21_ellipsis',true)) |  |
|  | S16 | TI (computer* or PC or “personal computer*” or “cell phone*” or “smartphone” or “smart phone*” or “mobile phone*” or (Tablet* and (device* or computer*)) or “web browser*” or website* or “mobile app”* or “computer program*” or “smartphone app*” or ios or android or ipad* or iphone* or ipod* or “computer assisted therapy*” or computer* or internet* or digital* or online* or deliver* or ((online or web*) and (education* or train*)) or “e health” or ehealth or mhealth or “m health” or telehealth or [...](javascript:showHistoryTerm('ctl00_ctl00_FindField_FindField_historyControl_HistoryRepeater_ctl22_ellipsis',true)) |  |
|  | S15 | S13 OR S14 |  |
|  | S14 | AB ("Mobile Applications") |  |
|  | S13 | TI ("Mobile Applications") |  |
|  | S12 | S10 OR S11 |  |
|  | S11 | AB ((MH "Computers, Hand-Held+") OR (MH "Computers, Portable+") OR (MH "Software") ) |  |
|  | S10 | TI ((MH "Computers, Hand-Held+") OR (MH "Computers, Portable+") OR (MH "Software") ) |  |
|  | S9 | S7 OR S8 |  |
|  | S8 | AB ((MH "Restricted Diet") OR (MH "Eating Behavior+") OR (MH "Eating Disorders Management (Iowa NIC)") ) |  |
|  | S7 | TI ((MH "Restricted Diet") OR (MH "Eating Behavior+") OR (MH "Eating Disorders Management (Iowa NIC)") ) |  |
|  | S6 | S4 OR S5 |  |
|  | S5 | AB ((“eating disorder*” or anorexi* or “bing* eat*” or bulimi* or OSFED* or "Other specified feeding or eating disorder*" OR (“bing* N2 eat*”) OR “appetite disorder*” or “feeding disorder*” OR (“disorder* N2 eat*”) OR ((Compulsiv*) N2 (eat* or vomit*)) (Topic) OR overeat* or “over-eat*” (Topic) OR (restrict* eat*) OR induce* vomit*) ) |  |
|  | S4 | TI ((“eating disorder*” or anorexi* or “bing* eat*” or bulimi* or OSFED* or "Other specified feeding or eating disorder*" OR (“bing* N2 eat*”) OR “appetite disorder*” or “feeding disorder*” OR (“disorder* N2 eat*”) OR ((Compulsiv*) N2 (eat* or vomit*)) (Topic) OR overeat* or “over-eat*” (Topic) OR (restrict* eat*) OR induce* vomit*) ) |  |
|  | S3 | S1 OR S2 |  |
|  | S2 | AB (MH "Eating Disorders+") OR (MH "Eating Disorders Management (Iowa NIC)") OR (MH "Binge Eating Disorder") OR (MH "Feeding and Eating Disorders of Childhood") OR (MH "Bulimia Nervosa") OR (MH "Avoidant Restrictive Food Intake Disorder") OR (MH "Bulimia") OR (MH "Anorexia") OR (MH "Anorexia Nervosa") |  |
|  | S1 | TI (MH "Eating Disorders+") OR (MH "Eating Disorders Management (Iowa NIC)") OR (MH "Binge Eating Disorder") OR (MH "Feeding and Eating Disorders of Childhood") OR (MH "Bulimia Nervosa") OR (MH "Avoidant Restrictive Food Intake Disorder") OR (MH "Bulimia") OR (MH "Anorexia") OR (MH "Anorexia Nervosa") |  |

***Ethos***

Eating AND computer
