# Supplementary material for: User Experiences of and Preferences for Self-Guided Digital Interventions for the Treatment of Mild to Moderate Eating Disorders: Systematic Review and Metasynthesis
Source: JMIR Ment Health. 2025 Jan 3;12:e57795. doi: 10.2196/57795 (PMC11748441; doi:10.2196/57795)
Supplement: Multimedia Appendix 3 [file mental_v12i1e57795_app3.docx]

**Multimedia Appendix 3**

**Main Findings for Individual Studies.**

*Main Findings for Individual Studies.*

| Authors: Publication year; Country | Main findings; User experiences | | Main findings; User preferences |
| --- | --- | --- | --- |
|  |  | |  |
| Jafari  2021  USA | Enthusiastic and open to trying a new approach. The app improved user’s wellbeing by promoting self-care activities. Users found the app’s convenience dependent on the context and environment. | | More interactive features to sustain engagement, a calorie counting function, and more social interaction.  Students rarely pays for apps and often overlook apps that cost money. |
| Yim and colleagues  2020  United Kingdom | The online nature made it easy to implement into daily life. Users experienced helpful reflections on their cognitions, emotions, interpersonal relations and behaviours.  Some thought it was not useful or relevant due to intersectionality and diversity among those with EDs.  Users also had mixed attitudes regarding the therapist-user interaction, and had ranging engagement styles. | | More tailored and personalised journey within the intervention, especially in adjusting notifications and reminders preferences, and option to use bookmark functions.  Users enjoyed the multimedia content to suit different learning styles.  Some desired downloadable or offline materials. |
| Juarascio and colleagues  2015  USA | Many users were enthusiastic and interested in using the app, especially regarding alerts and customisability.  Users were concerned about the app’s time demands, particularly with daily entries. | | Most users were comfortable sharing information with clinicians, but some preferred an option to not share data with clinicians.  Reminders and alerts were desired and seen as helpful in promoting change.  Users saw the app’s comprehensiveness as desirable, as it would allow users to track all behaviours in one place. |
| McClay and colleagues  2013  United Kingdom | Users felt positive towards the intervention, due to a desire for privacy and discretion.  Users noticed improvements in bulimic symptoms and behaviours towards food and eating.  Users valued the involvement of a support worker as a source of reassurance and motivation.  Some users had technical difficulties, resulting in feeling unsupported and unmotivated. | | More contact with the support worker to increase motivation.  Users highlighted privacy and autonomy as a positive aspect of the intervention. |
| Linardon and colleagues  2022  Australia | Users found the app straightforward to use, and thought the app had good interactivity.  Users believed the apps content was supported by evidence. Some thought the content was organised clearer than previous interventions they had tried.  Users agreed many activities were interactive, personalised and engaging, which aided motivation. | Users had mixed rating on visual design. They appreciated simplicity but preferred to have less colours.  More guidance, contact or reciprocity from a human. | |
| Yim and colleagues  2021  UK | Participants saw digital interventions as easy to integrate into daily life.  Most did not see self-help as a replacement for face-to-face treatment, instead a source of immediate support. | | Participants highlighted the importance of taking small steps towards recovery, and feedback on progress. |
| Jarman and colleagues  2022  Australia | Reflective questioning was seen as a key process in recovery. | | Preferred content that focuses on reflection to identify unhelpful thoughts and behaviours.  Desired social connection to facilitate hope and support through recovery.  Participants emphasised the importance of usability and language, especially readability and app accessibility, for promoting engagement. |
| Nitsch and colleagues  2016  USA | Users thought the program was easy and intuitive to navigate. Most enjoyed the layout.  Many disliked the wording of the exercise “ Dear Thighs”, and wording of assessment results.  Users had mixed views on the length of text. Some thought the questions were too long, this was an inconvenience. | | Users enjoyed the holistic approach and the focus on positive psychology and self-awareness.  Users would prefer shorter and clearer content, and avoidance of strong or serious language.  Users would prefer more personal and tentative feedback. |
